# Supplementary material for: Dynamics of Fanconi anemia protein D2 in association with nuclear lipid droplet formation
Source: J Cell Sci. 2025 Nov 13;138(21):jcs264430. doi: 10.1242/jcs.264430 (PMC12669972; doi:10.1242/jcs.264430)
Supplement: Supplementary information [file joces-138-264430-s1.pdf]

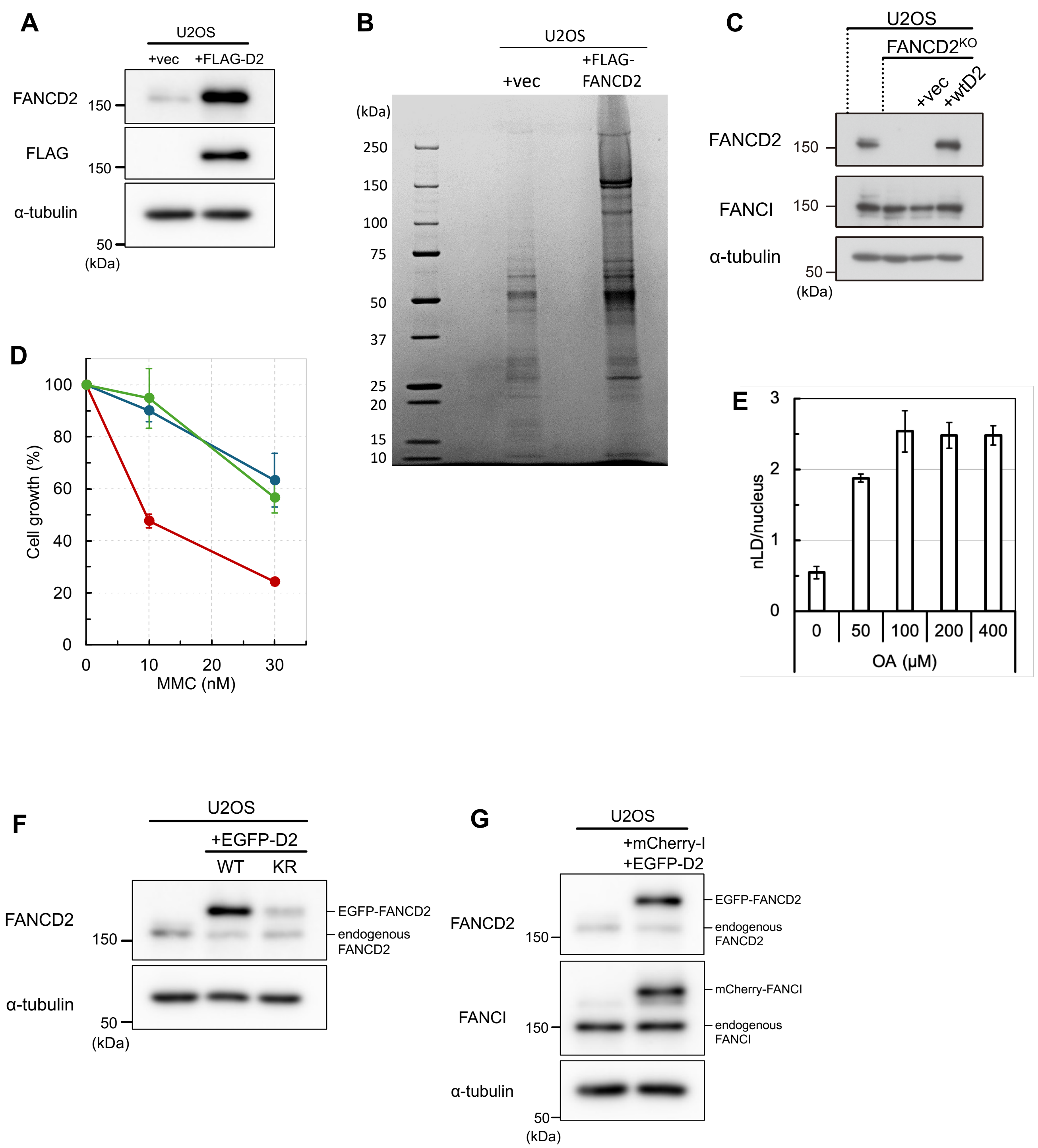

Fig. S1.

(A) Immunoblot analyses confirming the presence of endogenous FANCD2 and ectopically expressed FLAG-FANCD2. **(B)** The FANCD2 complex was immunoprecipitated from U2OS cells expressing FLAG-FANCD2. CBB staining is shown. **(C)** Immunoblot analyses of FANCD2 knockout (FANCD2^{KO}) and ectopically expressed FLAG-FANCD2 (wtD2). **(D)** FANCD2^{KO} cells (red) showed higher sensitivity to the DNA crosslinker mitomycin C (MMC) than the parental U2OS cells (blue). Expression of wild-type FANCD2 in the FANCD2^{KO} cells (green) restored MMC resistance equivalent to that of the parental U2OS cells. **(E)** Bar

Journal of Cell Science • Supplementary information

graph showing the number of nuclear lipid droplet (nLD) per nucleus. U2OS cells were treated with the indicated concentration of OA for 2 days. **(F)** Immunoblot analyses of EGFP-FANCD2 (WT) and mutant EGFP-FANCD2 (KR) in U2OS cells. **(G)** Immunoblot analyses confirming mCherry-FANCI and EGFP-FANCD2 in U2OS cells.

Journal of Cell Science • Supplementary information

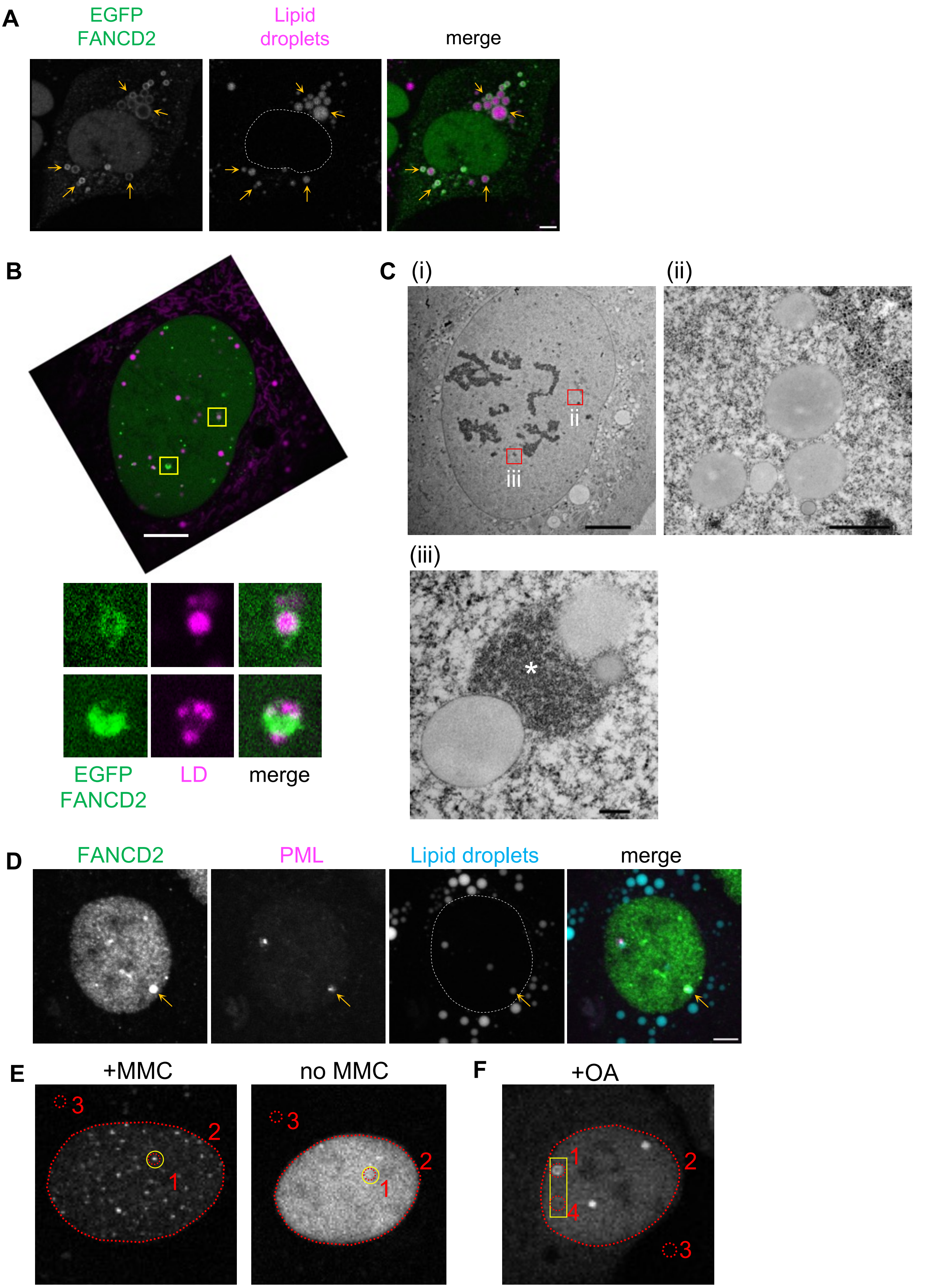

Yellow circle: bleached area
Red circle #1: FANCD2
#2: whole nucleus
#3: background
Relative EGFP intensity = (1-3)/(2-3)

Yellow rectangle: bleached area
Red circle #1: FANCD2 with nLD
#2: whole nucleus
#3: background
#4: FANCD2 without nLD
Relative EGFP intensity = (1-3)/(2-3) or (4-3)/(2-3)

Fig. S2.

(A) Localization of EGFP-FANCD2 in cells cultured with 200 µM of oleic acid (OA) for 2 days. Lipid droplets (LDs) were stained with Lipi-Blue. A dashed line indicates the location of FANCD2 (same as nucleus). Scale bar, 5 µm. (**B**, **C**) Correlative light–electron microscopy. U2OS cells stably expressing EGFP-FANCD2 were treated with 100 µM of OA for 24 h. (**B**) Cells were weakly fixed, and fluorescence images were captured first. Some nuclear LDs (nLDs; magenta) were surrounded by FANCD2 (green). Highly magnified images (yellow square) are shown below. The upper and lower panels correspond with C (ii) and (iii), respectively. Scale bar, 10 µm. (**C**) (i) Electron microscopy images of the cell shown in (**B**). (ii) and (iii) Highly magnified images (red squares) are shown. Scale bars, 10 µm (i), 1 µm (ii), and 200 nm (iii). The asterisk indicates the condensed chromatin area. (**D**) Localization of endogenous FANCD2 and PML in U2OS cells cultured with 200 µM of OA for 2 days. Orange arrows indicate an nLD with FANCD2 and PML. Representative images are shown. A dashed line indicates the location of FANCD2 (same as nucleus). Scale bar, 5 µm. **(E, F)** The relative EGFP-FANCD2 intensity in the FRAP analysis was calculated using the indicated formulas. The yellow circles and rectangle indicate the bleached areas. The numbers in panels E and F indicate the following: (**E**) 1, FANCD2; 2, whole nucleus; 3, background and (**F**) 1, FANCD2 with LD; 2, whole nucleus; 3, background; 4, FANCD2 without LD.

Journal of Cell Science • Supplementary information

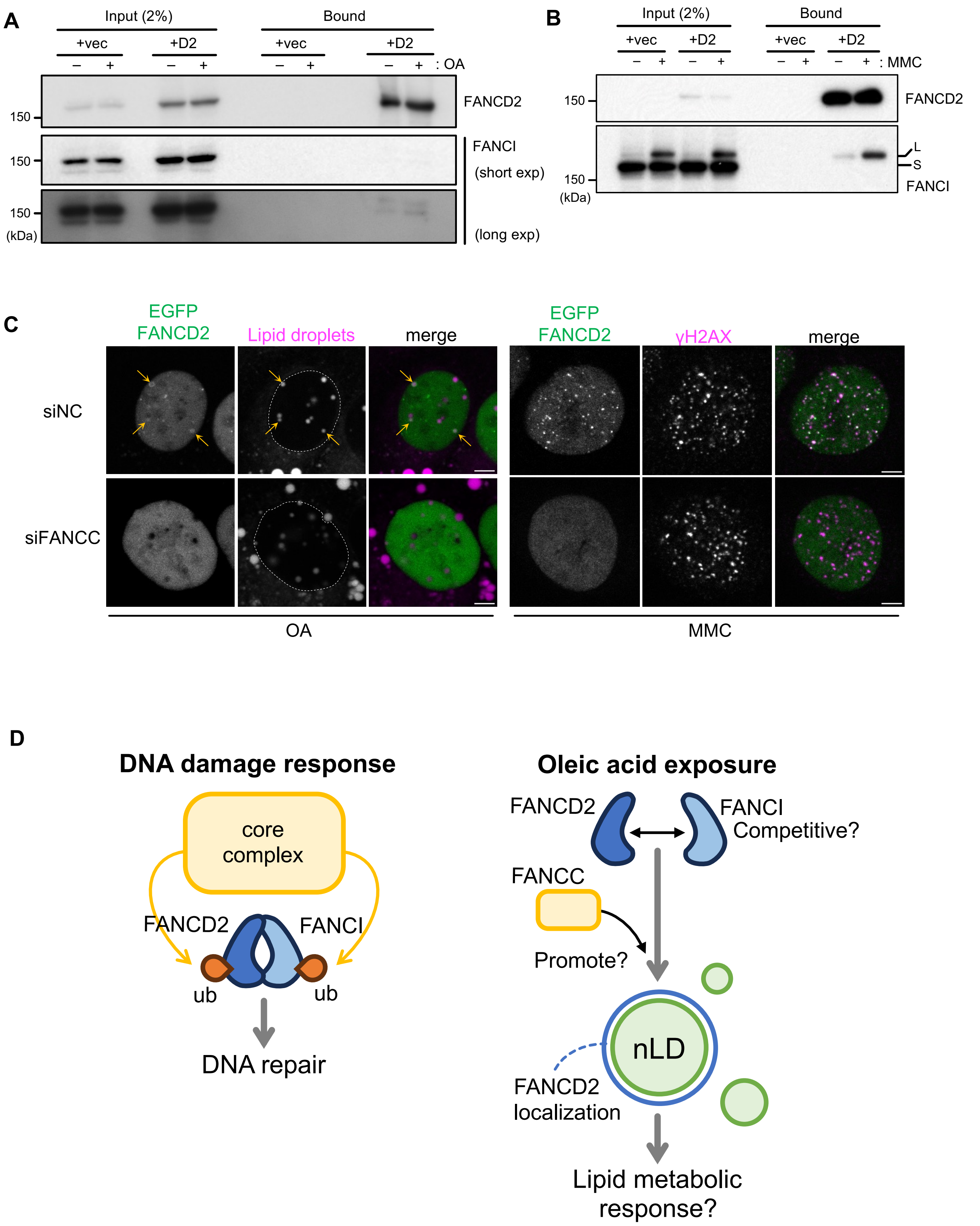

Journal of Cell Science • Supplementary information

Fig. S3.

(A, B) Co-immunoprecipitation assays were performed using U2OS cells stably expressing FLAG-FANCD2. Total cell extracts were prepared from cells treated with 200 μM oleic acid (OA) for 2 days or 1 μM mitomycin C (MMC) overnight and subjected to immunoprecipitation with anti-FLAG beads. **(B)** L and S indicate the monoubiquitinated and unmodified forms of FANCI, respectively. **(C)** Live-cell imaging (OA) and immunofluorescence (MMC) of U2OS cells expressing EGFP-FANCD2. The conditions for oleic acid (OA) and mitomycin C (MMC) treatment were the same as those described in Fig. 3C. Lipid droplets (LDs) were stained with Lipi-Deep Red. Scale bar, 5 μm. A dashed line indicates the location of FANCD2 (same as nucleus). **(D)** Canonical DNA damage response of FANC proteins (Left). Upon the DNA damage such as interstrand crosslink, the Fanconi anemia core complex ubiquitinates the ID complex to promote downstream DNA repair process. (Right) Hypothetical model in lipid metabolic response of FANCD2. Upon the OA exposure, FANCD2 is localized to nLDs in a FANCC-dependent manner. FANCI is a competitive factor for the nLD localization of FANCD2.

From Fig. 3A (γH2AX)

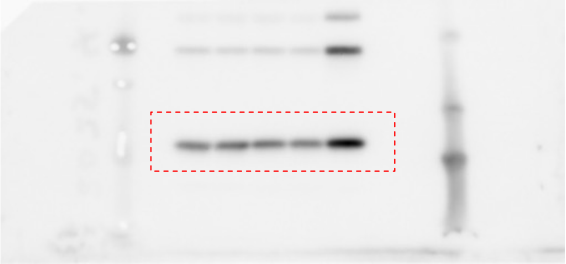

From Fig. 3A (Lamin B1)

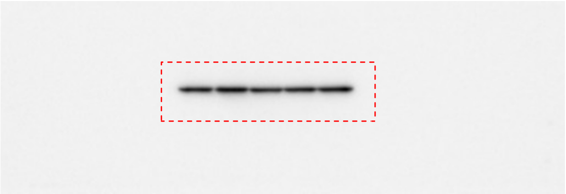

From Fig. 4C (FANCD2)

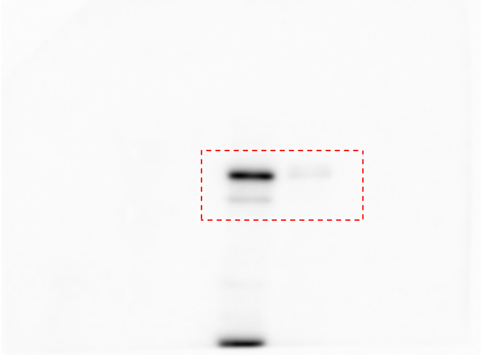

From Fig. 4C (FANCI)

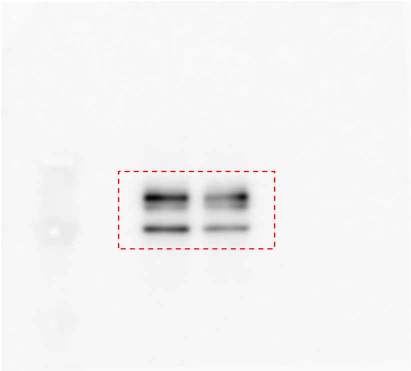

From Fig. 4C (α-tubulin)

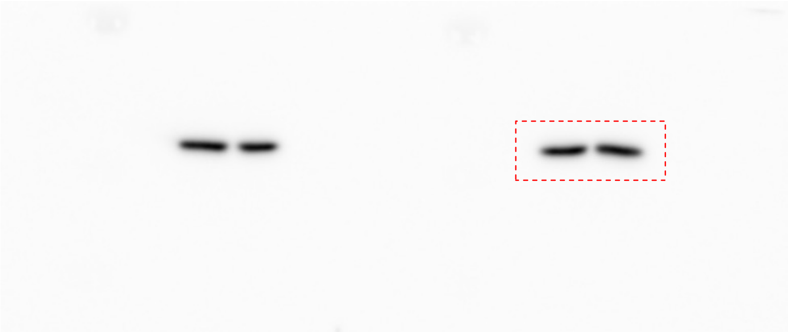

From Fig. 3B (FANCD2)

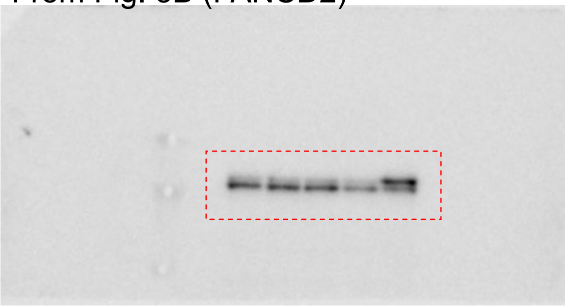

From Fig. 3B (α-tubulin)

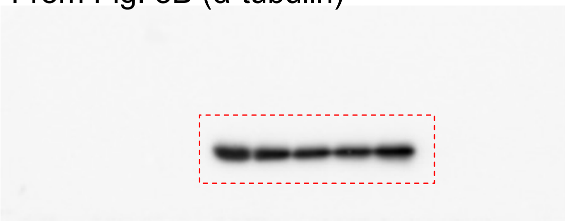

From Fig. 4E (FANCC)

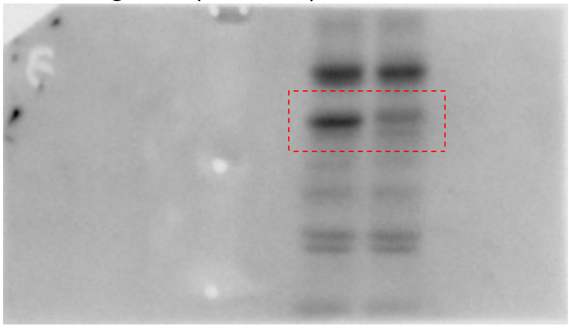

From Fig. 4E (FANCD2)

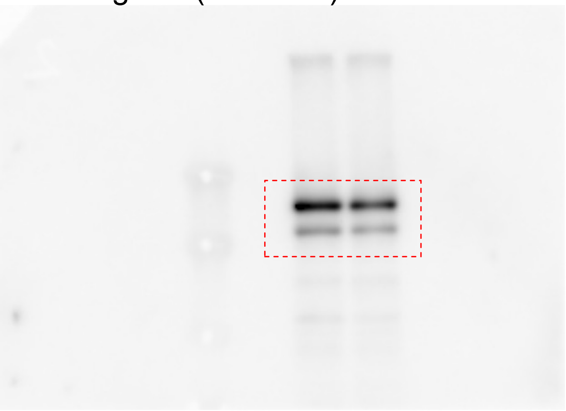

From Fig. 4E (α-tubulin)

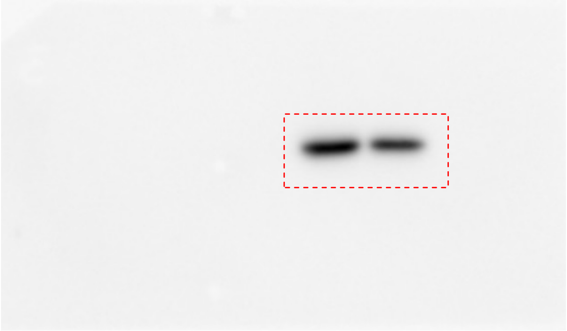

Fig. S4. Blot transparency

Journal of Cell Science • Supplementary information

Table S1. Candidates of FANCD2-interacting proteins

Available for download at
https://journals.biologists.com/jcs/article-lookup/doi/10.1242/jcs.264430#supplementary-data

Table S2. Proteomic analysis of FANCD2 complex in U2OS cell, related to Fig. 1A

Available for download at
https://journals.biologists.com/jcs/article-lookup/doi/10.1242/jcs.264430#supplementary-data

Table S3. Raw data of cellular fatty acid from Fig. 1B

Available for download at
https://journals.biologists.com/jcs/article-lookup/doi/10.1242/jcs.264430#supplementary-data

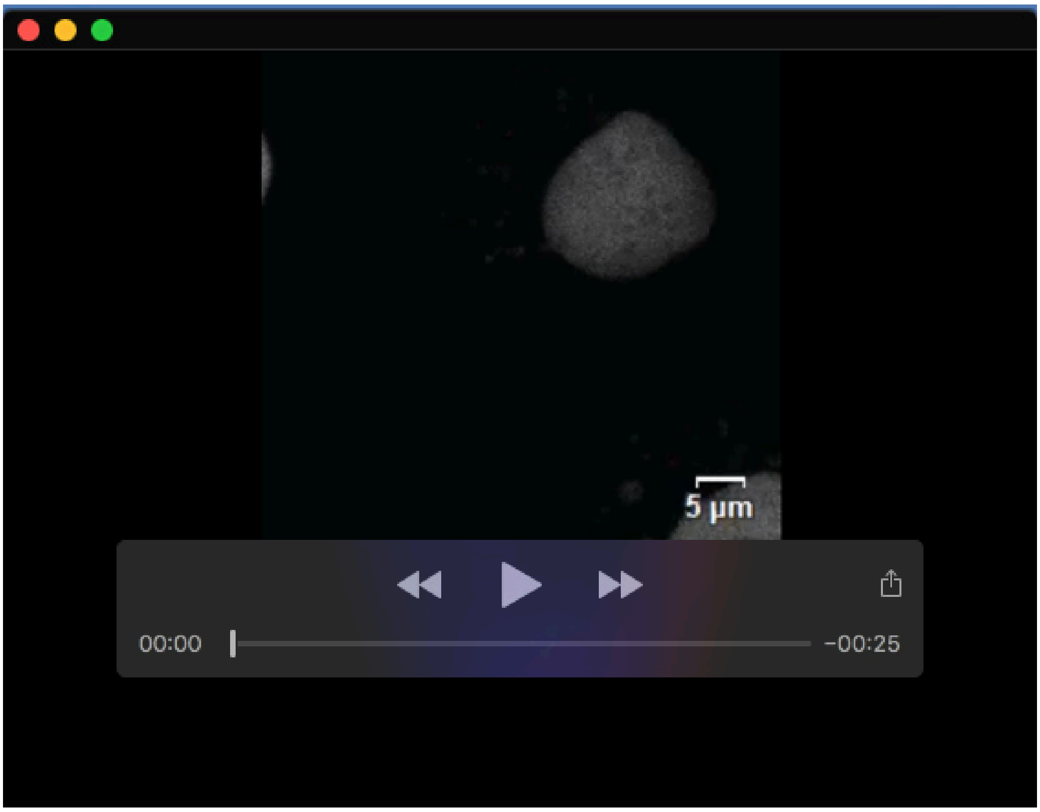

Movie 1. FANCD2 gathering to nLD are shown. EGFPFANCD2-expressing U2OS was treated with 200 µM oleic acid (OA) for 24 h. One frame was captured every 5 min. Scale bar, 5 µm. Selected frames are shown in Fig. 2A,B.

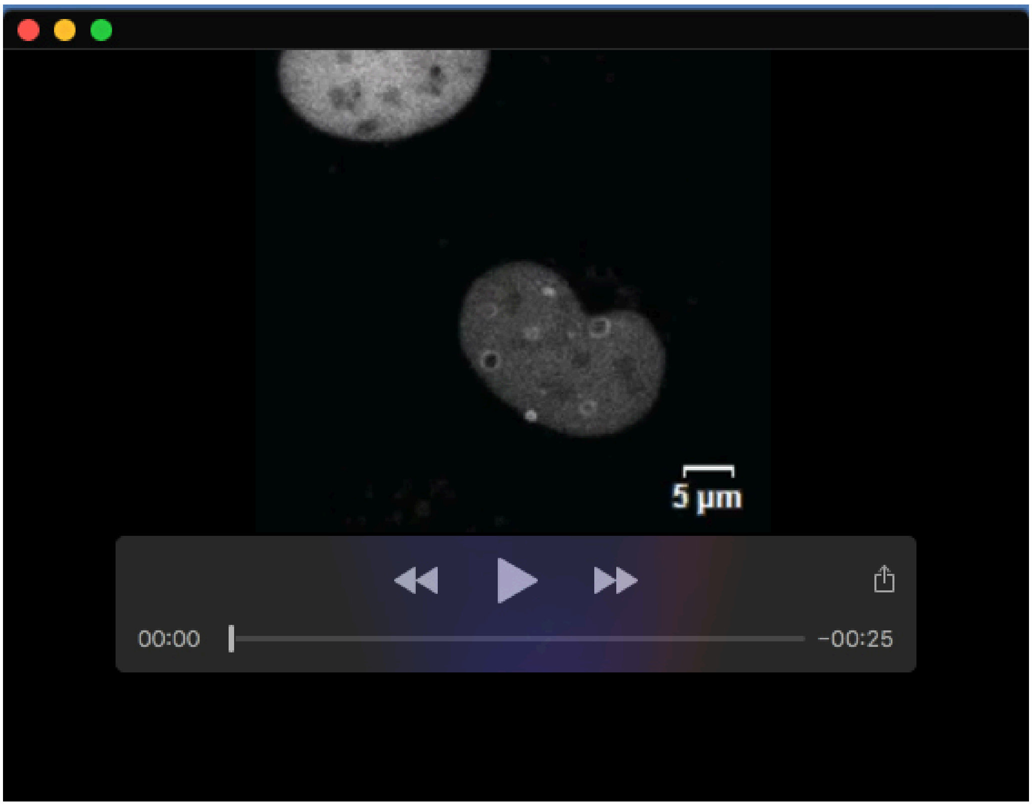

Movie 2. FANCD2 disappearing from nLD are shown. EGFPFANCD2-expressing U2OS was treated with 200 µM oleic acid (OA) for 24 h. One frame was captured every 5 min. Scale bar, 5 µm. Selected frames are shown in Fig. 2A,B.

Journal of Cell Science • Supplementary information

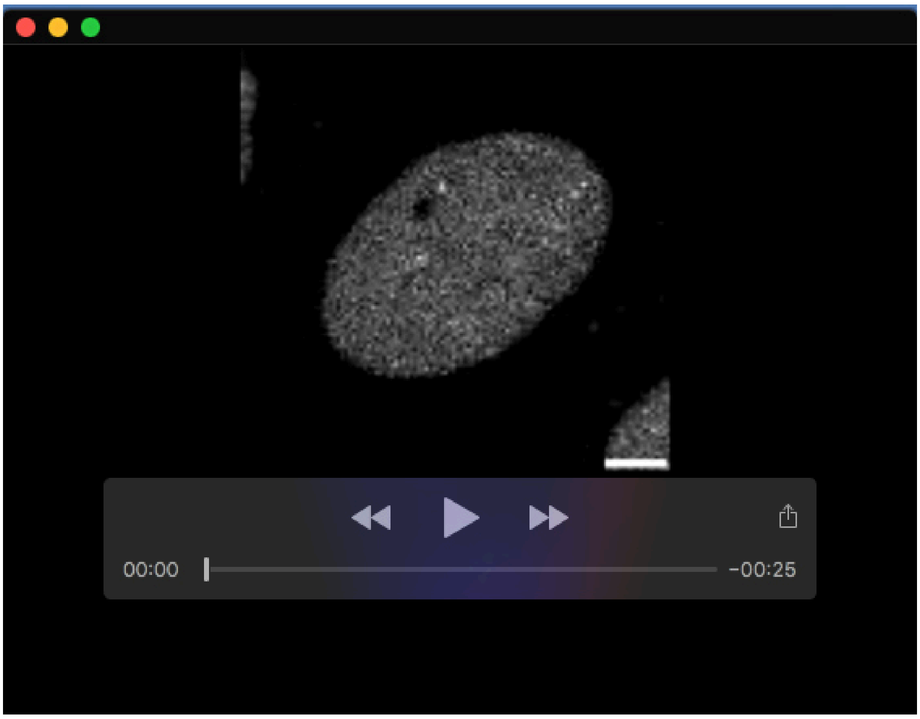

Movie 3. Representative movies of FRAP analyses are shown. One frame was captured every 10 sec. Scale bar, 5 µm. Selected frames are shown in Fig. 5A. Control.

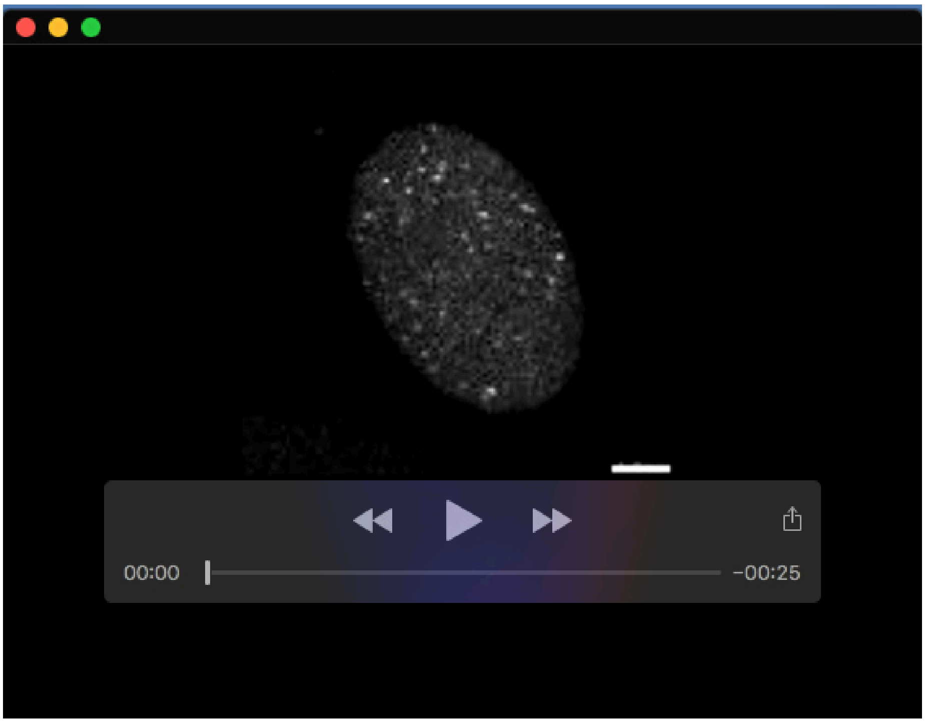

Movie 4. Representative movies of FRAP analyses are shown. One frame was captured every 10 sec. Scale bar, 5 µm. Selected frames are shown in Fig. 5A. MMC.

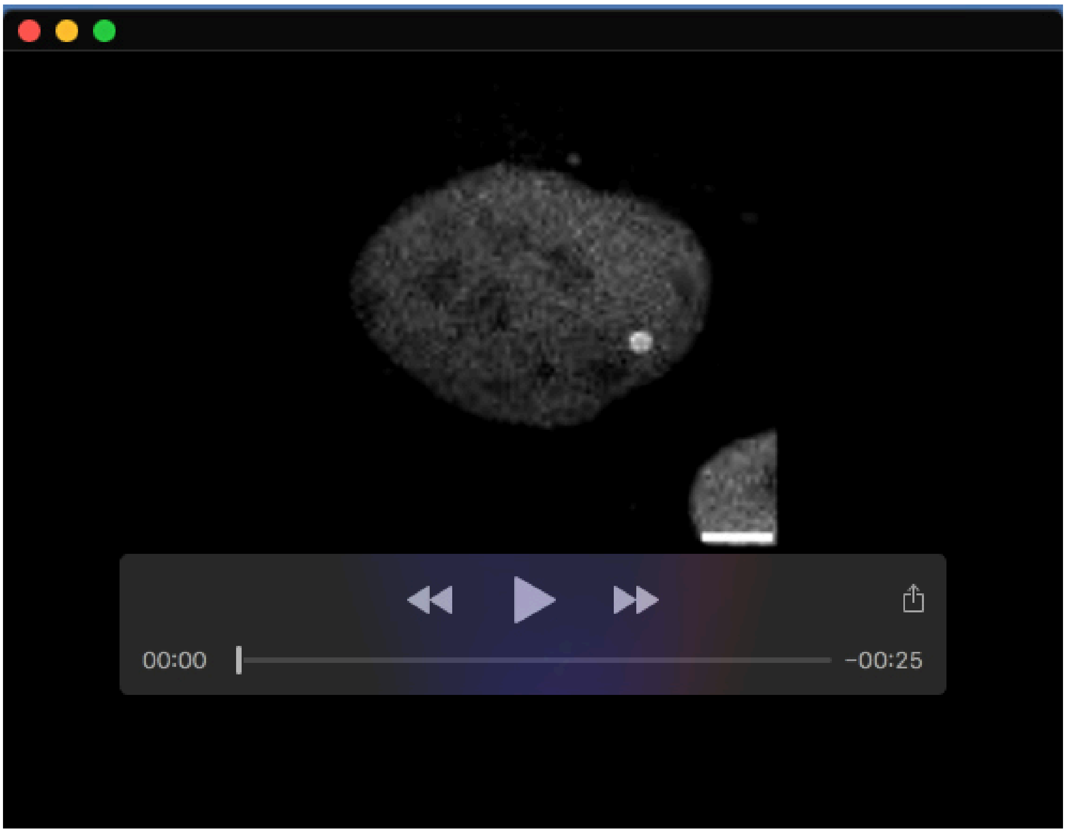

Movie 5. Representative movies of FRAP analyses are shown. One frame was captured every 10 sec. Scale bar, 5 µm. Selected frames are shown in Fig. 5A. OA.
